# Disparities in Receipt of Eye Exams Among Medicare Part B Fee-for-Service Beneficiaries with Diabetes — United States, 2017

**DOI:** 10.15585/mmwr.mm6845a3

**Published:** 2019-11-15

**Authors:** Elizabeth A. Lundeen, John Wittenborn, Stephen R. Benoit, Jinan Saaddine

**Affiliations:** ^1^Division of Diabetes Translation, National Center for Chronic Disease Prevention and Health Promotion, CDC; ^2^National Opinion Research Center, University of Chicago, Illinois.

Approximately 30 million persons in the United States have diabetes.* Persons with diabetes are at risk for vision loss from diabetic retinopathy and other eye diseases (*1*). Diabetic retinopathy, the most common diabetes-related eye disease, affects 29% of U.S. adults aged ≥40 years with diabetes (*2*) and is the leading cause of incident blindness among working-age adults (*1*). It is caused by chronically high blood glucose damaging blood vessels in the retina.^†^ Annual dilated eye exams are recommended for persons with diabetes because early detection and timely treatment of diabetic eye diseases can prevent irreversible vision loss^§^^,^^¶^ (*3*,*4*). Studies have documented prevalence of annual eye exams among U.S. adults with diabetes (*5*,*6*); however, a lack of recent state-level data limits identification of geographic disparities in adherence to this recommendation. Medicare claims from the 50 states, the District of Columbia (DC), Puerto Rico, and U.S. Virgin Islands (USVI) were examined to assess the prevalence of eye exams in 2017 among beneficiaries with diabetes who were continuously enrolled in Part B fee-for-service insurance, which covers annual eye exams for beneficiaries with diabetes.** This report also examines disparities, by state and race/ethnicity, in receipt of eye exams. Nationally, 54.1% of beneficiaries with diabetes had an eye exam in 2017. Prevalence ranged from 43.9% in Puerto Rico to 64.8% in Rhode Island. Fewer than 50% of beneficiaries received an eye exam in seven states (Alabama, Alaska, Kentucky, Louisiana, Nevada, West Virginia, and Wyoming) and Puerto Rico. Non-Hispanic white (white) beneficiaries had a higher prevalence of receiving an eye exam (55.6%) than did non-Hispanic blacks (blacks) (48.9%) and Hispanics (48.2%). Barriers to receiving eye care (e.g., suboptimal clinical care coordination and referral, low health literacy, and lack of perceived need for care) might limit Medicare beneficiaries’ ability to follow this preventive care recommendation. Understanding and addressing these barriers might prevent irreversible vision loss among persons with diabetes.

This analysis was performed using 100% of the Centers for Medicare & Medicaid Services research identifiable files but was restricted to claims for Medicare beneficiaries continuously enrolled in Part B fee-for-service for all of 2017.^††^ Part B covers outpatient services, including ophthalmologic services. This analysis includes Medicare beneficiaries aged ≥65 years, as well as those aged <65 years who qualify through disability or disease status, in the 50 U.S. states, DC, Puerto Rico, and USVI. Analyses were conducted using SAS Enterprise Guide (version 9.4; SAS Institute).

The outcome measure was the prevalence among Medicare Part B fee-for-service beneficiaries with diabetes of receiving an eye exam during January–December 2017. Beneficiaries received a diagnosis of diabetes if they had at least one diagnosis code (*International Classification of Diseases*, *Tenth Revision*) or procedure code (Current Procedural Terminology [CPT] and Healthcare Common Procedure Coding System) defined in the Chronic Conditions Data Warehouse diabetes algorithm on at least one claim during 2016–2017.^§§^ Prevalence was calculated as the number of continuously enrolled beneficiaries with diabetes who had an eye exam claim in 2017 divided by the number of continuously enrolled beneficiaries with diabetes in that year. Eye exams were defined using CPT codes 92002, 92004, 92014, and 92014 and other evaluation and management visit CPT codes if the provider taxonomy codes indicated an eye care provider.^¶¶^ Unadjusted percentages are presented nationally and by state and race/ethnicity (white, black, Hispanic, Asian/Pacific Islander, American Indian/Alaska Native, and other). Age-standardized estimates, using direct standardization, were similar, and these data are not presented. Statistical testing was not performed because these data represent 100% of Medicare beneficiaries who met the inclusion criteria.

Among the 30,238,300 continuously enrolled Medicare Part B fee-for-service beneficiaries in 2017, a total of 8,341,000 (28%) had a diabetes diagnosis. The majority (72.4%) of these beneficiaries with a diabetes diagnosis were aged 65–84 years, with fewer aged 40–64 years (14.6%) or ≥85 years (12.1%). Overall, 73.3% of these beneficiaries were white, 13.0% were black, 8.3% were Hispanic, 3.5% were Asian/Pacific Islander, 0.8% were American Indian/Alaska Native, and 1.0% were other racial/ethnic groups.

Nationally, 54.1% of beneficiaries with diabetes had an eye exam in 2017 (Table). The prevalence ranged from 43.9% in Puerto Rico to 64.8% in Rhode Island. In seven states (Alabama, Alaska, Kentucky, Louisiana, Nevada, West Virginia, and Wyoming) and Puerto Rico, <50% of beneficiaries with diabetes received an eye exam (Table) (Figure 1). In nine states (Connecticut, Delaware, Hawaii, Iowa, Maine, Massachusetts, Nebraska, North Dakota, and Rhode Island) ≥60% of beneficiaries with diabetes had an eye exam in 2017.

**TABLE:** Percentage of Medicare Part B fee-for-service beneficiaries with diagnosed diabetes who had an eye exam in 2017, by state and race/ethnicity* — Medicare Part B fee-for-service claims data, 2017

State	No.	Racial/Ethnic group, %						
		All	White	Black	Hispanic	Asian/Pacific Islander	American Indian/Alaska Native	Other
Alabama	159,300	**47.1**	48.5	43.0	41.2	49.8	54.0	48.3
Alaska	15,500	**47.5**	47.8	50.2	45.0	46.2	46.3	47.3
Arizona	137,000	**55.6**	56.9	49.3	48.6	56.0	56.2	58.3
Arkansas	103,200	**52.4**	53.4	47.2	46.0	51.8	52.2	52.2
California	707,600	**51.5**	52.8	44.6	47.1	56.8	45.5	54.8
Colorado	77,000	**52.5**	54.5	47.5	44.2	53.0	48.9	54.1
Connecticut	93,400	**62.3**	63.9	57.6	54.6	59.9	58.9	59.6
Delaware	42,800	**60.4**	61.2	58.2	55.4	61.2	58.3	65.5
District of Columbia	16,100	**51.6**	56.7	50.7	49.9	56.6	—^†^	55.7
Florida	569,900	**56.6**	58.5	50.2	49.3	54.9	53.0	58.9
Georgia	238,600	**50.4**	52.1	46.4	43.2	50.3	35.2	54.8
Hawaii	27,100	**63.1**	58.8	50.2	57.2	65.1	54.2	64.2
Idaho	38,900	**51.7**	52.3	40.0	45.7	50.4	44.7	52.6
Illinois	356,500	**54.2**	55.4	49.5	49.9	58.7	45.0	58.2
Indiana	207,200	**51.6**	52.4	45.3	44.3	53.1	50.4	54.6
Iowa	101,200	**64.7**	65.3	53.9	53.8	55.5	45.1	69.1
Kansas	92,000	**59.3**	60.5	50.8	48.8	56.9	49.1	61.5
Kentucky	156,400	**47.7**	47.6	48.9	44.0	52.0	42.3	51.9
Louisiana	136,000	**49.2**	49.9	47.8	47.9	45.5	44.8	52.4
Maine	44,000	**60.7**	60.8	51.2	61.7	59.9	50.9	59.2
Maryland	205,800	**53.4**	55.4	49.6	50.6	56.0	43.0	56.5
Massachusetts	183,400	**64.4**	65.2	61.5	60.2	60.8	55.8	65.1
Michigan	303,000	**53.3**	54.9	46.6	49.5	55.2	46.0	54.9
Minnesota	66,300	**58.1**	59.5	47.9	52.0	49.4	53.4	51.2
Mississippi	127,300	**50.3**	51.8	47.7	47.4	44.0	51.0	53.2
Missouri	175,500	**53.4**	54.1	48.4	50.0	53.1	39.7	52.3
Montana	27,500	**54.9**	56.2	47.3	47.1	58.0	43.0	55.1
Nebraska	55,700	**60.1**	61.2	52.4	48.7	56.0	38.8	57.7
Nevada	62,500	**48.8**	50.1	43.4	44.3	50.8	51.6	53.9
New Hampshire	45,200	**55.6**	55.7	55.0	50.2	55.8	50.0	54.4
New Jersey	305,000	**53.9**	55.7	48.0	47.2	55.8	42.8	57.3
New Mexico	53,600	**50.9**	52.8	49.4	45.2	58.4	60.3	50.9
New York	513,800	**58.5**	59.9	54.7	52.5	59.2	50.4	59.5
North Carolina	314,400	**54.4**	55.9	51.0	50.0	52.8	45.7	55.3
North Dakota	20,000	**64.3**	65.3	52.5	53.5	60.0	53.1	66.7
Ohio	303,100	**52.7**	53.1	49.3	47.9	57.3	38.5	57.2
Oklahoma	136,900	**50.9**	50.8	47.7	43.2	49.2	55.5	54.2
Oregon	74,500	**54.2**	54.5	55.8	50.0	55.1	50.5	59.2
Pennsylvania	320,100	**57.1**	58.5	47.9	47.3	53.6	40.8	57.8
Rhode Island	21,400	**64.8**	65.9	59.5	53.9	59.8	56.4	64.7
South Carolina	173,900	**53.5**	55.2	49.0	48.2	54.2	43.2	55.8
South Dakota	24,700	**58.3**	60.1	43.6	50.2	56.6	43.1	67.0
Tennessee	190,400	**50.5**	51.5	45.2	46.3	46.3	44.2	49.3
Texas	582,200	**51.1**	53.6	45.2	47.5	51.9	54.2	55.1
Utah	44,400	**53.7**	54.7	46.7	44.9	47.7	45.4	50.9
Vermont	21,300	**57.3**	57.4	44.0	54.0	53.5	64.3	60.3
Virginia	260,600	**56.9**	58.3	53.2	50.0	55.9	46.9	61.3
Washington	156,000	**54.9**	55.8	49.0	48.6	54.5	45.5	57.5
West Virginia	79,100	**46.2**	46.2	45.2	42.8	51.8	44.4	47.3
Wisconsin	126,400	**58.0**	59.1	47.9	50.0	49.5	53.9	59.4
Wyoming	16,700	**49.7**	50.6	46.4	46.4	44.6	29.3	48.8
Puerto Rico	25,200	**43.9**	49.5	—^†^	43.9	—^†^	—^†^	—^†^
U.S. Virgin Islands	5,500	**54.9**	49.0	56.6	44.6	56.4	—^†^	52.9
**Total**	**8,341,000**	**54.1**	**55.6**	**48.9**	**48.2**	**56.3**	**51.9**	**57.2**

* Whites, blacks, Asian/Pacific Islanders, American Indians/Alaska Natives, and Others were non-Hispanic; Hispanic persons could be of any race.

^†^ Data were suppressed because of small sample size, defined as either 1) a denominator <11 or 2) a numerator <3 and denominator <30.

**FIGURE 1:**
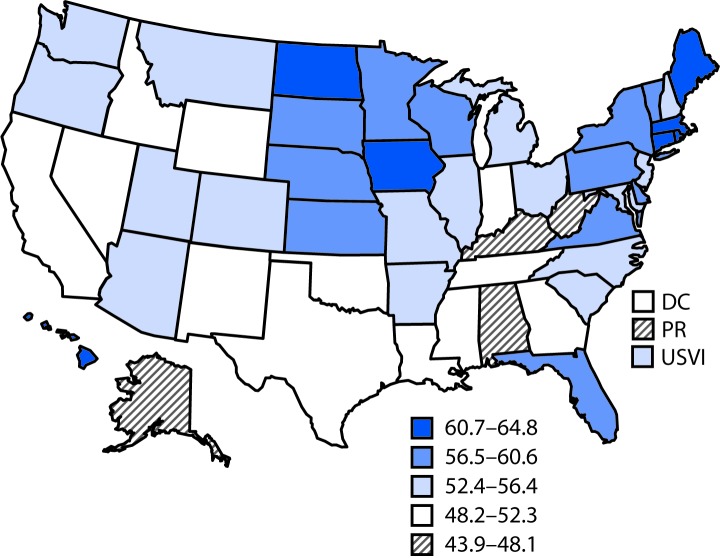
Percentage of Medicare Part B fee-for-service beneficiaries with diabetes who had an eye exam, by state — United States, 2017 **Abbreviations:** DC = District of Columbia; PR = Puerto Rico; USVI = U.S. Virgin Islands.

Nationally, the prevalence of having an eye exam was lower among Hispanic (48.2%) and black (48.9%) beneficiaries with diabetes than it was among whites (55.6%). This was also observed in 46 states and DC. Prevalence was higher among beneficiaries aged ≥85 years (58.6%) and 65–84 years (56.9%) than among those aged 40–64 years (38.0%) or 18–39 years (31.7%) (Figure 2).

**FIGURE 2:**
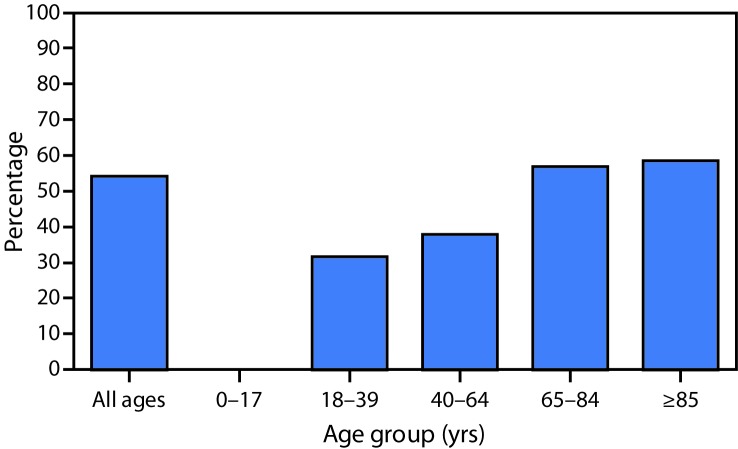
Percentage of Medicare Part B fee-for-service beneficiaries with diabetes who had an eye exam, by age group* — United States, 2017 * Data for beneficiaries aged 0–17 years were suppressed because of small sample size (≤100).

## Discussion

This report of recent state-level prevalence of receiving an eye exam among Medicare Part B fee-for-service beneficiaries with diabetes found that, although Medicare covers annual eye exams for beneficiaries with diabetes, only 54.1% of these beneficiaries received an eye exam in 2017. Among Hispanic and black beneficiaries and those in seven states, <50% of beneficiaries received an eye exam.

These findings are consistent with those from other studies. An analysis of the 2005–2008 National Health and Nutrition Examination Survey data found that 51.2% of adults aged ≥40 years with diabetes had an eye exam in the past year (*5*). A study of claims for U.S. patients aged 10–64 years with commercial or employer-sponsored health insurance found that among persons with diabetes and no diabetic retinopathy, 48.1% had not received an eye exam during the 5-year study period and 15.3% had an annual or biennial exam (*6*).

Dilated eye exams are an important preventive care practice for early detection of diabetic retinopathy. Seventy-three percent of persons with diabetic retinopathy are unaware of their disease (*7*). Early detection and timely treatment can prevent irreversible vision loss. The efficacy and cost-effectiveness of diabetic retinopathy screening among persons with diabetes is well established (*4*), and professional organizations recommend annual screening. The American Diabetes Association recommends that persons with diabetes have annual eye exams, with consideration of biennial exams if there is no evidence of retinopathy on at least one annual eye exam and blood glucose is controlled (*3*).

Studies have documented enablers and barriers to obtaining regular eye exams. A study using a small sample of Medicare beneficiaries aged ≥65 years found that 37% had an eye exam at least once every 15 months during a 5-year period (*8*). Factors associated with more frequent eye exams included older age, being married, higher educational attainment, and a higher score on the Charleson Comorbidity Index (which predicts mortality for a patient with a range of comorbid conditions) (*8*). Factors associated with lower frequency of eye exams included being male, living ≥20 miles from an ophthalmologist, low cognitive function, and limitations in instrumental activities of daily living (skills and abilities needed to perform certain day-to-day tasks associated with living independently). A study of adults with diabetes in 22 states found that the factors most commonly cited for not seeking annual eye care were not perceiving a need for care and cost or lack of insurance; other factors included a lack of transportation, distance to an eye doctor, and not having or knowing of an eye doctor (*9*). These findings highlight a lack of perception of the need for eye care and geographic and transportation barriers. Telemedicine might be a promising health care innovation to address geographic barriers in accessing eye care professionals for diabetic retinopathy screenings (*10*). Through following evidence-based recommendations and providing patient education, health care providers can play an important role in improving the rate of receipt of annual eye exams among persons with diabetes. In addition, optimizing systems for eye care referrals and reminders (e.g., clinical decision support tools in electronic health records) and improving care coordination between clinicians managing diabetes and those providing eye care might address barriers attributable to low patient awareness.

The findings in this report are subject to at least four limitations. First, some beneficiaries who had eye exams might be nonadherent with recommendations; claims provide insufficient detail to identify dilated eye exams. Second, patients might have multiple insurers, and services reimbursed by a supplemental plan would not be recorded in Medicare claims, thereby underestimating eye exam prevalence. Third, Medicare data do not include care provided by the Indian Health Service; therefore, the data presented are likely not representative of the American Indian/Alaska Native population. Finally, this analysis excluded the 33.9% of Medicare beneficiaries enrolled in Medicare managed care plans.***

Although annual eye exams are covered for all Medicare Part B fee-for-service beneficiaries with diabetes, only approximately half of these beneficiaries received an eye exam in 2017. Geographic and racial/ethnic disparities in adherence to this preventive care practice were identified. This low prevalence of receipt of annual eye exams could have significant implications for vision loss from diabetes-related eye diseases. CDC’s Vision and Eye Health Surveillance System, which provides data on U.S. vision and eye health conditions and use of eye care, is an important tool to identify trends and assess eye health disparities among persons with diabetes.^†††^ These data can be used to inform strategies and interventions to prevent vision loss among Medicare beneficiaries with diabetes.

**Summary**

**What is already known about this topic?**

Annual eye exams are an important preventive care practice for persons with diabetes. Early detection and treatment of diabetic retinopathy and other eye diseases can prevent irreversible vision loss.

**What is added by this report?**

Nationally, 54.1% of Medicare Part B fee-for-service beneficiaries with diabetes had an eye exam in 2017. Disparities by state and race/ethnicity were identified.

**What are the implications for public health practice?**

Although Medicare covers annual eye exams for beneficiaries with diabetes, the prevalence of receipt of exams is low. Interventions are needed to improve adherence to annual eye exams to prevent irreversible vision loss among persons with diabetes.

## Footnotes

* https://www.cdc.gov/diabetes/pdfs/data/statistics/national-diabetes-statistics-report.pdf.

† https://www.nei.nih.gov/learn-about-eye-health/eye-conditions-and-diseases/diabetic-retinopathy.

§ https://www.aao.org/eye-health/tips-prevention/top-five-diabetes-steps.

¶ http://aoa.uberflip.com/i/374890-evidence-based-clinical-practice-guideline-diabetes-mellitus.

** https://www.medicare.gov/coverage/eye-exams-for-diabetes.

†† https://www.cdc.gov/visionhealth/vehss/data/claims/medicare.html.

§§ https://www2.ccwdata.org/web/guest/condition-categories.

¶¶ http://www.norc.org/PDFs/VEHSS/VEHSSClaimsRegistryAnalysisPlan.pdf.

*** https://www.cms.gov/Research-Statistics-Data-and-Systems/Statistics-Trends-and-Reports/CMSProgramStatistics/2017/Downloads/MDCR_ENROLL_AB/2017_CPS_MDCR_ENROLL_AB_1.pdf.

††† https://www.cdc.gov/visionhealth/vehss/index.html.
